# Implementing Policy, Systems, and Environmental Change Through Community Coalitions and Extension Partnerships to Address Obesity in Rural Louisiana

**DOI:** 10.5888/pcd17.190284

**Published:** 2020-02-27

**Authors:** Denise Holston, Jessica Stroope, Melissa Cater, Michelle Kendall, Stephanie Broyles

**Affiliations:** 1 Louisiana State University Agricultural Center, School of Nutrition and Food Sciences, Baton Rouge, Louisiana; 2 Louisiana State University Agricultural Center, Northeast Region, Winnsboro, Louisiana; 3 Louisiana State University Health Sciences Center, School of Public Health, New Orleans, Louisiana; 4 Pennington Biomedical Research Center, Baton Rouge, Louisiana

## Abstract

Community coalitions and agents funded by the Louisiana State University Agricultural Center’s Healthy Communities program implemented multilevel obesity prevention interventions in 3 rural parishes (ie, counties) with an obesity prevalence of 40% or higher. The Healthy Communities coalitions appraised local health concerns through needs assessments and community forums. On the basis of local needs and the evidence base, the coalitions identified and implemented policy, systems, and environmental (PSE) strategies and supporting education to promote healthy behavior change among residents, overcoming barriers in the process. Interventions varied by parish but included Complete Streets implementation plans, healthy retail initiatives, play space improvements, downtown beautification projects, and Smarter Lunchrooms.

**Summary**

**What is already known about this topic?**

Policy, systems, and environmental (PSE) change strategies combined with education are known to support healthy behavior change and obesity prevention in a community-based setting.

**What is added by this report?**

We share the process through which coalitions in rural Louisiana implemented PSE change.

**What are the implications for public health practice?**

By understanding the process, potential barriers, and time needed to establish and create fully operational community coalitions, communities can implement strategies to improve community engagement and entities funding PSE work can set better grantee expectations, ultimately leading to more sustainable obesity prevention interventions.

## Background

Louisiana has an obesity prevalence of 36.8%, making it the state with the fourth highest prevalence in the nation; 43.5% of black adults and 32.9% of white adults are affected (1). Obesity is concentrated in rural areas, where 6 rural parishes (ie, counties) have a prevalence of 40% or higher (2,3). Adults and children living in rural settings have higher rates of nutrition-related chronic diseases (4), lower diet quality (5), and lower physical activity levels compared with urban residents (6). Behaviors that reduce obesity and chronic disease risk are challenging to adopt in rural areas because of local nutrition and physical activity environments (7). Thus, positive behavior changes are unlikely if left unsupported by the environments in which individuals live, work, and play (8).

The Louisiana State University Agricultural Center (LSU AgCenter), part of the Louisiana Cooperative Extension Service, has faculty in all 64 parishes of the state, providing evidence and practice-based information to local communities through informal education and outreach opportunities. Because behavior change is unlikely to result from education alone and must be supported by environmental change, the cooperative extension service now directs nutrition agents to prioritize policy, systems, and environmental (PSE) change strategies, supported by education efforts (9).

In 2015–2018, through a cooperative agreement with the Centers for Disease Control and Prevention (CDC) High Obesity Program, the LSU AgCenter planned and implemented Healthy Communities, a multilevel, community-led approach to obesity prevention in 3 rural parishes in Louisiana (Madison, Tensas, and St. Helena) with adult obesity rates higher than 40% (Table). Local Extension agents worked with community coalitions to identify and implement PSE change strategies to address the rural nutrition and physical activity environment, with the ultimate goal of promoting healthy behaviors among residents and improving obesity rates and quality of life. Coalitions also prioritized opportunities for education to provide additional support for healthy behavior change.

**Table:** Descriptive Characteristics of Target Counties, Healthy Communities Obesity Initiative, Rural Louisiana, 2015–2018

Characteristic	Louisiana Parish		
	Madison	Tensas	St. Helena
Population, n^a^	11,803	4,893	10,714
Obesity prevalence, %^b^	41.9	40.8	41.7
Poverty, %^a^	39.6	33.8	27.6
African American, %^a^	63.1	54.1	52.3

a Source: 2016 American Community Survey.

b Source: 2014 Funding opportunity announcement (FOA) DP14–1416: programs to reduce obesity in high obesity areas.

## Partnerships and Collaborations

The LSU AgCenter, through the CDC High Obesity Program grant, provided Healthy Communities Cooperative Extension agents in 3 target parishes. These agents recruited community members to form a Healthy Communities coalition for each parish. Coalition members included representatives from schools, local government, churches, businesses, nonprofit organizations, and health care, as well as youth and community residents. Members contributed to the coalitions through collaborative planning, leveraging resources such as donations from businesses, publicity, volunteer labor, in-kind donations, and raising awareness of community health needs through social and professional networks. The direction of the coalitions was determined through collaboration among 153 participants via 4 community forums. Forum attendees shared information and perspectives on the assets and health challenges present in the community and discussed the changes that would be the most effective and easiest to implement. The community forums served to build awareness, inform needs assessments, and gather community support. Guided by input from the community forum, baseline assessments, and the PSE evidence base, the local coalitions identified the most relevant, achievable, and significant PSE changes for their communities. When disagreements arose between coalition members, the extension agents were able to redirect coalition members to the needs prioritized by community members in the community forum. Referring back to the forum provided a neutral way to work through conflict.

Additionally, the extension team contacted CDC for assistance navigating cultural sensitivity. Residents of all 3 parishes were mostly black (Table), and in 2 parishes white coalition members expressed racial stereotypes and prejudices. One member complained in a semi-structured interview that Healthy Communities had become a “black project.” Although all projects were designed to reach the entire community, the farmers market was hosted by a historically black church, which may have been viewed by this interviewee as only benefiting black residents. In another parish, white coalition members raised concerns about planned park improvements, fearing “they would just trash [the park] again,” with “they” functioning as reference to previous park-users, most of whom were black. Although black coalition members framed concerns differently, the coalition as a whole expressed concerns about crime. To address concerns about potential park neglect, content experts from Texas A&M specializing in Crime Prevention Through Environmental Design worked with community members, coalition members, the police department, and the parish facilities manager to create a plan to help the park thrive. The plan included hosting regular park events that were used to further community forum priorities. In addition to a CDC cultural sensitivity Skype workshop with the Healthy Communities agents, a trainer from the University of Maryland was brought in to address cultural competency with all field faculty. Additionally, future coalition member surveys will collect demographic data and assess perceived level of voice in decision making.

In addition to these barriers, poverty and collaboration instability were obstacles within our partnerships and collaborations. One parish had 4 mayors in 18 months, which significantly slowed progress. Developing key stable partnerships in the parish, such as with the police jury (ie, the Louisiana equivalent to a county board of commissioners) was instrumental in maintaining project progress. Some problems were connected to vacant buildings; discerning who owned the buildings took considerable time, beyond the project funding timeframe. All parishes raised concerns about funding; change of any kind, even with grant support, seemed unrealistic to many community members at the project’s outset. Part of the work of each coalition included establishing trust and building confidence that change could be possible.

## Multilevel Community Obesity Prevention Program

Community-based obesity prevention programs that use education and outreach coupled with PSE change strategies can improve obesity-related behaviors among residents (10). These approaches supplement and go beyond individual or group educational strategies used by health educators as part of a multicomponent, multilevel collaborative program delivery model. Residents may need introduction to a PSE change through an education program or event to adopt and sustain a new behavior. In terms of obesity prevention, education combined with PSE change strategies is more effective, economical, and sustainable than using either strategy alone (11).

The overall project timeline included needs-assessment activities conducted in target parishes, community forums and coalition formation, and public project kick-off implemented (year 1); policy discussion and change, selection of systems, and environmental interventions by coalitions for their communities (year 2); and implementation of environmental interventions and collection of follow-up assessments (year 3). Ongoing technical assistance was provided to coalitions during meetings and events throughout the project duration. The implemented PSE strategies varied somewhat by community but included park revitalization, stenciling play spaces, healthy retail initiatives, Smarter Lunchroom initiatives, community and school gardens, initiating and supporting farmers markets, downtown beautification projects, Complete Streets demonstrations, and Complete Streets rural plan development.

To promote physical activity for children, each grant parish created multicolor painted play spaces. Multicolor painted markings are a low-cost PSE change that increases physical activity among children (12). The grant provided paint and stencils; the coalitions chose locations likely to have a significant impact and recruited volunteers to paint the play space. To highlight this change, the St. Helena Parish coalition planned a children’s yoga event at the newly painted play space.

Each parish partnered with a local grocery store or corner store to increase the availability of healthy options. Stores were provided promotional materials and technical assistance to increase the visibility and availability of whole grain foods and fresh fruits and vegetables, which increases fruit and vegetable purchases (13). To draw attention to these changes, 2 coalitions planned cooking demonstrations showcasing improvements in availability of fresh fruits and vegetables.

People living in communities with built environments that support active-friendly routes to everyday destinations tend to be more physically active (14). Committing to Complete Streets implementation plans on the part of the communities was successful in part because no funds were needed. The parishes were not committing to build sidewalks; they were committing to consider the needs of pedestrians and people on bicycles in future projects. Community support was gathered through Complete Streets demonstrations days, which introduced residents to what an active transportation–promoting PSE change might look like. Technical assistance for demonstrations was provided by 3 planners from the Center for Planning Excellence, who spent 2 days setting up each Complete Street demonstration, assisted by coalition members. Community members were given opportunities to test proposed improvements, provide comments, and witness active transportation assets that could positively support local health behaviors. The demonstrations showed how low-cost changes can affect the core of a town (eg, adding a crosswalk), while also showing what more substantial changes (eg, sidewalks, pocket parks, bike lanes) could create. The Complete Street demonstrations were planned to avoid state-owned roads, which limited how projects could be prioritized. Some desired changes were not pursued because approvals for projects involving state-owned roads were not available. A member of the state extension office now has a seat on the state Complete Streets advisory council and is working to make permissions feasible for PSE changes in rural communities.

## Implications for Public Health

Healthy Communities models a multilevel, community-participatory obesity prevention intervention using a combination of PSE change and education. Gathering support through community forums and building community coalitions to drive PSE change are time-intensive activities. It takes significant work to build trust so that coalition members believe that their voices will be heard. The process has potential to bolster ongoing community support for changes (15). Addressing multiple levels and projects through the same coalition helps build support and awareness for all PSE changes. Coalitions can help redirect projects when insurmountable barriers arise, as well as navigate obstacles, even as facilitators must be prepared to help coalitions resolve intra-coalition conflict. Obesity has multifaceted causes and is best addressed at multiple levels through many approaches. Although achieving actual reductions in obesity rates takes time, pursing PSE changes while also providing education may be more effective than either strategy alone (10).

The Healthy Communities coalitions remain active and continue to work toward improving the health of their parishes through PSE changes. Some of the changes made — such as a Complete Streets plan — can continue to change the local environment as new construction takes into consideration active transportation. Rural communities may not have access to as many resources as urban counterparts but through Healthy Communities, local coalitions have been able to leverage community resources to make their communities as healthy as possible. The presence of extension agents helped to create and sustain momentum in substantial ways. The agents helped focus the coalitions, reminded members of meetings, brought in outside experts, resolved disagreements, arranged meeting spaces, ensured notes were taken and disseminated, and helped maintain community engagement. The agents continue to work with the coalitions to make progress on long-term goals, such as park improvements and increased food accessibility.

Because PSE change can take time, early successes are critical. To keep coalition members engaged, having multiple projects with varying timelines was important; there were enough projects for coalition members to see movement in at least one project aspect at all times but not so many that a coalition became exhausted. Maintaining a “future work” list may be an effective tool in providing assurance that ideas can be temporarily suspended and resumed at a future date, even as other projects are given precedence. Entities funding future PSE work should consider the length of proposed projects, including how much time should be allotted to detect individual behavior change before conducting a post evaluation. Future studies could include evaluations at multiple points to determine how much time is needed to detect behavior change and health improvements from PSE interventions, which is especially important for projects built through community coalitions. The time needed to establish a coalition, assess needs, and prioritize PSE change strategies results in initiating PSE projects approximately 1 year after coalitions are formed. Funding such projects for a longer period is needed to allow sufficient time to detect substantial evidence of PSE change effects on individual behavior and health.
